# HIV infection, seasonality and younger age predicting incident Bell’s palsy among black South Africans

**DOI:** 10.1186/s12883-020-01965-0

**Published:** 2020-10-21

**Authors:** Dali Magazi, Benjamin Longombenza, Siyazi Mda, Kees Van der Meyden, Marcus Motshwane, Mirabel Nanjoh, Olakunle Towobola

**Affiliations:** 1 Department of Neurology, Sefako Makgatho Health Sciences University, Pretoria, South Africa; 2 Department of Internal Medicine, University of Kinshasa, Kinshasa, Democratic Republic of Congo; 3 Department of Paediatrics, Sefako Makgatho Health Sciences University, Pretoria, South Africa; 4 Department of statistics, Tshwane University of technology, Pretoria, South Africa; 5 Faculty of health Sciences, Walter Sisulu University, Mthatha, South Africa; 6 Department of Internal Medicine, Sefako Makgatho Health Sciences University, Pretoria, South Africa

**Keywords:** Bell’s palsy, Seasons, HIV, Young age, Males, South Africa

## Abstract

**Background:**

Although South Africa (SA) is facing a high prevalence of HIV infection, there is no literature from this region on a link between Bell’s palsy and HIV. The aim of this study was to identify the occurrence of Bell’s palsy in relation to demographics, seasons and HIV status among black South Africans.

**Methods:**

This retrospective cohort was conducted among adult black patients, without Bell’s palsy in 2003, presenting to the neurology outpatients department at Dr. George Mukhari Academic hospital, Pretoria, South Africa, between 2004 (study baseline) and 2012 (end test). Gender, age, HIV status, and seasons were potential predictors of Bell’s palsy using Cox regression model and Kaplan Meier curves.

**Results:**

From the baseline of 1487 patients, 20.9% (*n* = 311) experienced Bell’s palsy onset by the end of the study. In univariate analysis, male gender (RR = 2.1 95% CI 1.7–2.5; *P* <  0.0001), age less than 30 years (RR = 2.9 95% CI 2.4–3.6; *P* <  0.0001), HIV seropositivity (RR =2.9 95% CI 2.3–4.9; *P* < 0.0001).

The highest incidence in winter (30.3% *n* = 136/450) vs. incidences during other seasons with Intermediate values during Summer (25.3% *n* = 136/450) and Autumn (20.7% *n* = 64/308) and the lowest incidence in Spring (23.7% *n* = 16/353) *P* < 0.0001) were predictors of Bell’s palsy. In multivariate analysis at adjusting for gender, the most significant and independent predictors of incident Bell’s palsy were HIV seropositivity (HR = 6.3 95% CI 4.8–8.3; *P* < 0.0001), winter (HR = 1.6 95% CI 1.2–2.1; *P* < 0.0001) vs. other seasons, and younger age < 30 years (HR = 7.1 95% CI 5.6–9.1; *P* < 0.0001) vs. older age groups.

**Conclusion:**

Seasonality, younger age and HIV positivity are important and independent risk factors of Bell’s palsy. Education and awareness programs on the possible effects of HIV and seasons on the development of Bell’s palsy are necessary. This would lead to a better understanding and even a possible development of avoidance measures for this condition amongst young black South Africans.

## Background

Bell’s palsy is an acute idiopathic lower motor neuron facial palsy, not associated with pyramidal signs. The seasonal occurrence of Bell’s palsy has been debated for more than two centuries, first suggested by Nicolaus Friedreich in 1798 [1]. This continues to be a matter for debate since there is no universal consensus as to whether there is even a link between Bell’s palsy and the seasons. An experimental study whereby maxillofacial tissue was frozen showed an immediate malfunction of the facial nerve which recovered after several weeks, the conclusion being that low temperatures trigger facial paralysis [2]. Indeed, in some countries, incident Bell’s palsy has been observed more during the cold months of winter than in the warm months of summer [3]. However, in European settings such as Turkey, Bell’s palsy was found to mostly occur in spring and least in the winter months [4]. There is no published data on the relationship between seasons and Bell’s palsy in Sub-Saharan Africa generally. HIV infection has however, been associated with Bell’s palsy in the tropical countries with no reports in this regard from the Sahel countries [5–7]. Our clinical impression was that there seems to be a link between certain seasons and a surge in the occurrence of Bell’s palsy in South Africa with its high prevalence of HIV infection. Therefore, the objective of this study was to determine whether demographics, seasons and HIV status among black South Africans play a role in the occurrence of Bell’s palsy.

## Methods

This retrospective cohort was conducted among adult black patients, without Bell’s palsy in 2003, presenting to the neurology outpatient department at Dr. George Mukhari Academic hospital, Pretoria, South Africa, between 2004 (study baseline) and 2012. Gender, age, HIV status, and seasons were potential predictors of Bell’s palsy. Bell’s palsy was defined by an acute idiopathic (unilateral/ bilateral) peripheral facial weakness with no pyramidal signs. Younger and older ages were defined by median age of 30 years. Cold seasons included autumn / fall and winter, while warm seasons included spring and summer. Autumn was defined as a transitional season from summer leading to winter while spring was defined as a transitional season from winter leading to summer.

### Statistical analysis

Categorical variables were presented as proportions (%), while the continuous variable (age) was expressed as the median. In univariate analysis, relative risk (RR) of incident Bell’s palsy and its 95% confidence interval (CI) was calculated (Mantel- Haenszel test). In multivariate analysis, the Cox regression model was computed to quantify the association (hazards ratio = HR with its 95% CI) between exposures to univariate potential risk factors, and the multivariate risk of Bell’s palsy after adjusting for confounding factors. Differences between exposed and non-exposed arms were investigated and Kaplan-Meier survival curves were generated using the log rank test. *P* - value < 0.05 was regarded as statistically significant. The statistical software IBM * SPSS version 22 for Windows (IBM, Chicago, IL, USA) was used for all analyses.

## Results

**Fig. 1:**
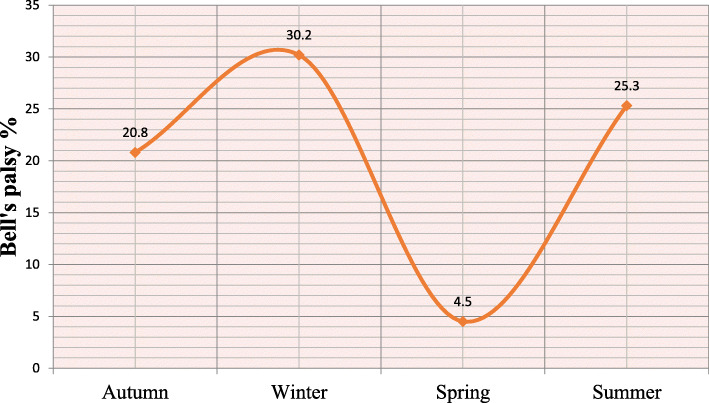
Proportion of patients with Bell’s palsy (%) in relation to the seasons

**Fig. 2:**
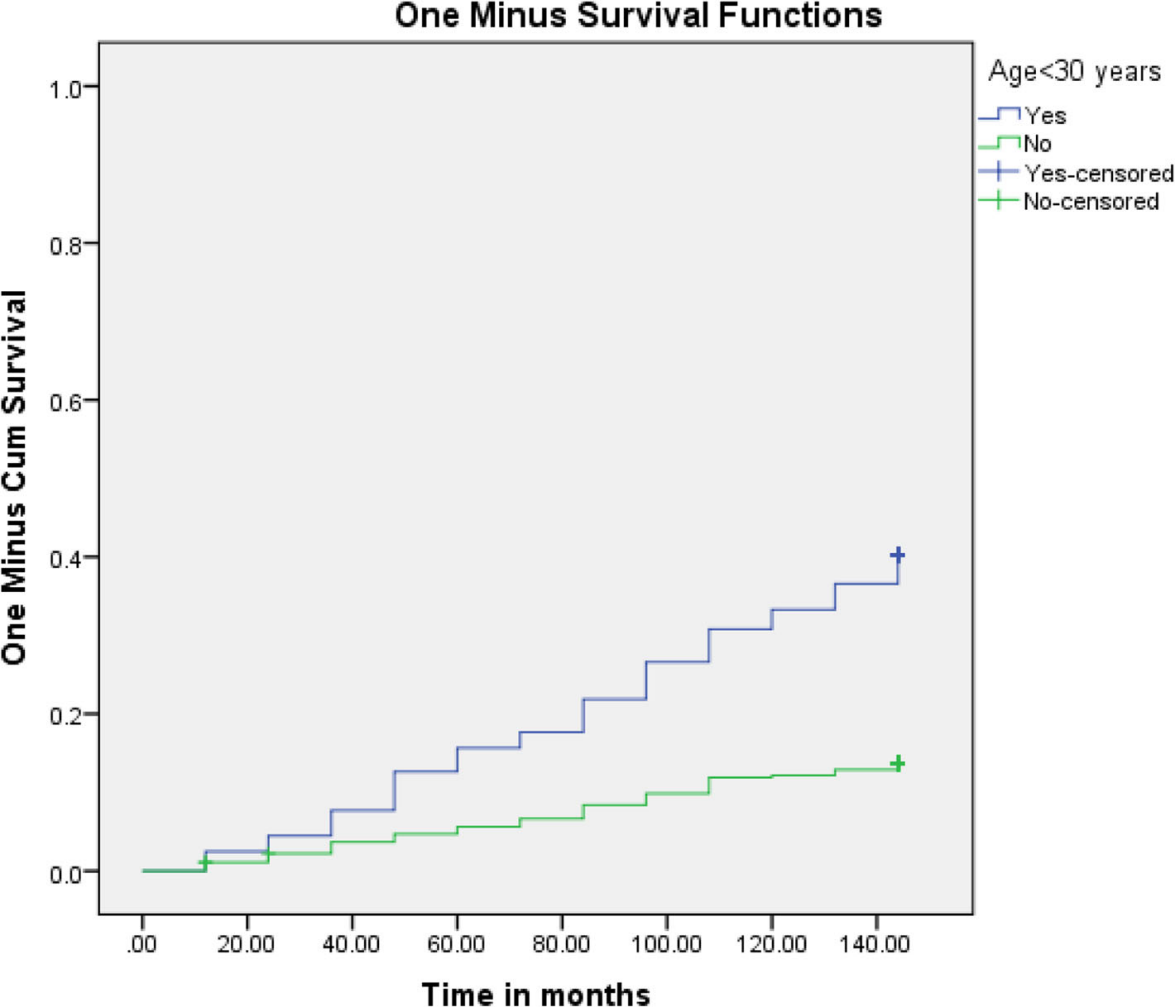
Survival function for Bel’s palsy incidence by age stratification

**Fig. 3:**
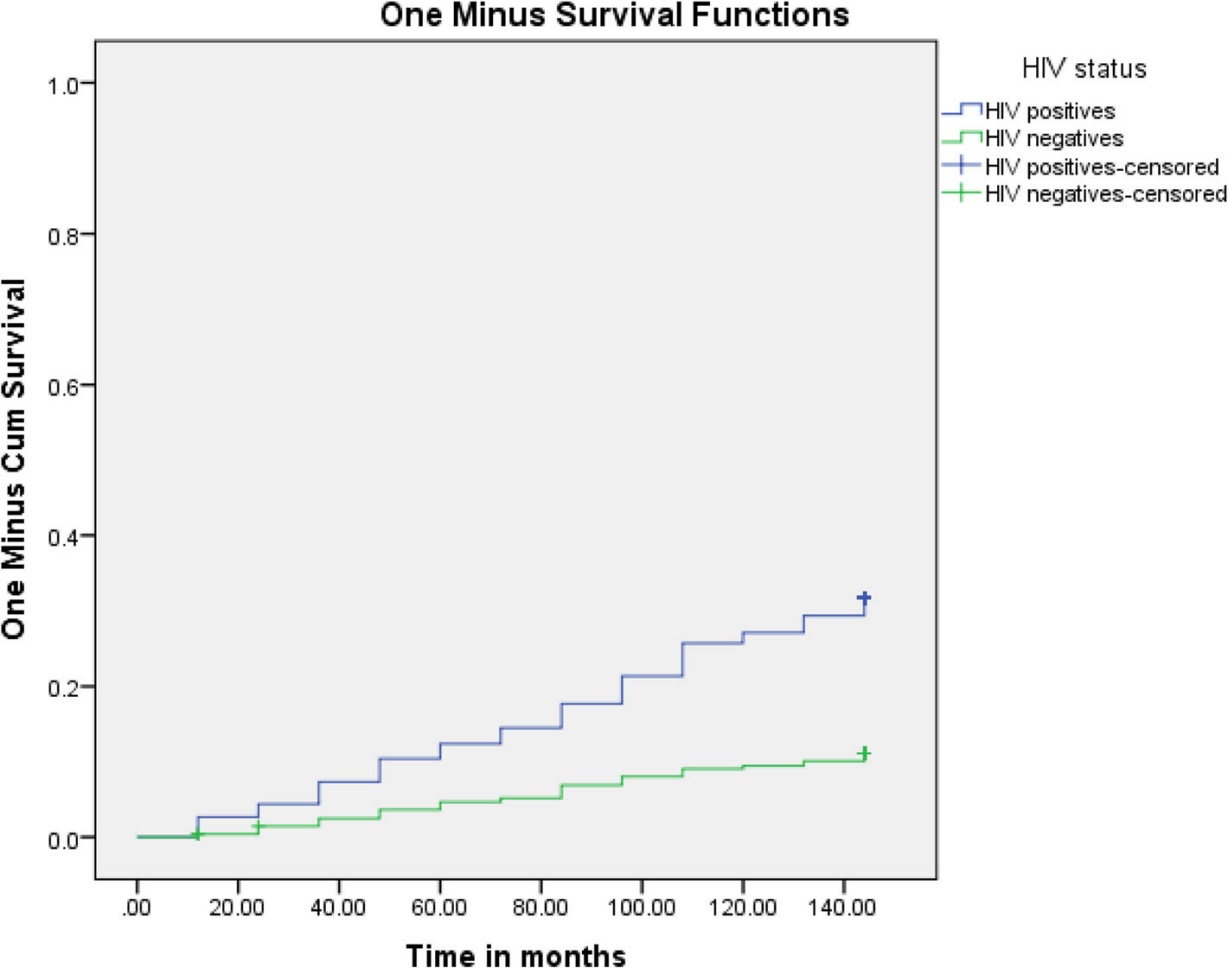
Survival function to Incidence of Bell’s palsy by HIV status

Table 1 shows a significant univariate association between male gender, younger age < 30 years, HIV seropositivity, seasonality and incident Bell’s palsy. There was a seasonal acrophase rhythm with a winter peak and nadir (trough) during spring, the second highest in Summer which in turn was followed by Autumn with intermediate incidences of Bell’s palsy (Fig. 1). In multivariate (Cox regression) analysis adjusting for gender, the most significant and independent predictors of incident Bell’s palsy were HIV seropositivity, winter season, and younger age < 30 years. The survival distributions for the different levels of age (Fig. 2) and HIV status (Fig. 3) using the log rank test are demonstrated on Kaplan Meier curves.

**Table 1:** Univariate association between potential predictors and Bell’s palsy.

Variable of interest	Incident n (%)	Bell’s palsy	***P***-value
		**RR (95% CI)**	
**Gender**			
**Males**	138/415 (33.3)	2.1 (1.7–2.5)	< 0.0001
**Females**	173/1072 (16.1)		
**Age groups**			
**< 30 years**	162/402 (40.3)	2.9 (2.4–3.6)	< 0.0001
**≥ 30 years**	149/1085 (13.7)		
**Seasons**			
**Autumn**	64/308	< 0.0001	
**Winter**	136/450		
**Spring**	16/353		
**Summer**	95/376		
**HIV**			
**Positive**	225/711 (31.6)	2.9 (2.3–3.6)	< 0.0001
**Negative**	86/776 (11.1)

A majority of HIV positive females with Bell’s palsy were not on ARV treatment (60%) whilst the inverse was true for males (60% on ARV treatment). There were no abnormalities of cognition demonstrated (including learning, memory and attention) in all patients with Bell’s palsy with no difference in mental performance between those on ARVs and ARV naïve patients.

## Discussion

The present study demonstrated univariate and multivariate associations between younger age, male gender, cold seasons, HIV infection, and incident Bell’s palsy among black South African patients. These specific findings confirmed or disputed data reported from the literature as will be further elaborated.

### Age and Bell’s palsy

Bell’s palsy can occur at any age but the question has been which age groups are most vulnerable to the condition. These vulnerable age groups could shed light further on the underlying pathophysiology of the condition. Our study showed a peak at the age less than 30 years. This is at variance with various previous studies in Europe and Asia which found Bell’s palsy to peak in the fourth and fifth decade [8–11].

A study from the Benghazi region of Libya showed people above the sixth decade in age as being prone to Bell’s palsy [3]. This was at variance with neighbouring Egypt whose findings showed the highest ages of involvement to be 40–49 years [12]. A door-door survey in the rural part of the Cordillera province of Bolivia however, found similarly to the Libyan study with the prevalence of Bell’s palsy increasing with age to peak at 65 years [13].

A Nigerian study showed the median age of occurrence to be 35.5 years, echoing studies in European settings [14]. A Togolese study of 150 individuals showed the average age of occurrence to be 31.4+/− 8.81 years [5]. A study with similar findings as our study was from Romania where the dominant age group of occurrence was 17–30 years [15].

The narrative on age from these studies, worldwide is that Bell’s palsy is a condition of the young and middle-aged adult with the elderly being in the minority. A Brazilian study of 180 patients with Bell’s palsy however, found two peaks, namely, third- fourth decade and sixth decade of life [16]. Some studies caution that when someone older than 60 years gets Bell’s palsy, a secondary cause like diabetes mellitus could be the reason [8]. What puts the young adult at more risk than other age groups has not been previously explained.

### Gender

There was a male preponderance on univariate analysis which however, didn’t emerge on using the multivariate analysis in this study. A majority of studies on Bell’s palsy demonstrate no difference in occurrence between the sexes [8, 17]. There are studies however, that demonstrated a difference in occurrence between the sexes with some showing a female preponderance whilst some show it to be mostly amongst males [11, 13]. Risky sexual behaviour (multiple partners, unprotected sex) and lifestyle such as heavy alcohol intake more common in South African males than females might also explain the univariate association between male gender and Bell’s palsy in this study [18].

### Seasons

Previous studies on the relationship between seasons and Bell’s palsy have had varied observations with some finding a clear association or seasonal clustering and yet others not finding any such connection. In a study of a 1000 patients with facial palsies, reporting on seasonal data between 1969 and 1973, no preference of occurrence was found between warm and cold climates [19]. This was echoed by a Danish study of 2500 patients with Bell’s palsy [8]. Yet another study from Greece found no relation to the occurrence of Bell’s palsy and weather [20]. A study of 1181 US soldiers with Bell’s palsy however, revealed a clear increase in occurrence during the cold months as opposed to the warm seasons [21]. This is echoed by a Libyan study in the region of Benghazi where they also found clustering in the December and January months [3]. The proponents of clustering in the months of winter suggest that this could be linked to a flare up of the herpes (HSV-1), a virus which has been associated with Bell’s palsy [21]. In Turkey, Bell’s palsy was found to be more frequent in Spring with the nadir in winter [4]. The non-uniform manifestations of global seasons and possible confounding factors like atmospheric pressure and humidity could account for the varied findings. The seasonal cyclical occurrence of Bell’s palsy demonstrated in the present study has never been described before.

### Possible mechanisms

Patients seen at our hospital are from a poorer section of Pretoria, a social construct from Apartheid South Africa. The low socioeconomic status would carry stressors unique to this population. A link between psychological stress and the development of Bell’s palsy has been described [22].

To our knowledge, this is the first study to demonstrate HIV infection, younger age and cold seasons as independent predictors of Bell’s palsy among black South Africans. HIV infection is associated with oxidative stress affecting both the central and peripheral nervous systems (albeit with the latter being a result of some antiretroviral drug regimes) [23–25]. The excess free radicals formed lead to tissue injury with resultant impairment of function. Oxidative stress has also been described with the herpes group of viruses [26]. Furthermore, cold weather has also been associated with oxidative stress which is a possible reason for a surge in the occurrence of Bell’s palsy during colder seasons [27]. The free radicals formed with oxidative stress lead to tissue damage and could possibly be a factor in accelerating incident Bell’s palsy in these black South Africans.

HIV infection has been associated with lower levels of S-adenosylmethionine, the principal methyl donor in cytosine methylation metabolite important for transmethylation which is important for myelin formation [28]. Low levels of this metabolite can result in an aberrant DNA methylation pattern and development of neuropathies [29].

The extent to which nutrition played a role was not established in this population. Vitamin deficiencies have been described as a common co-existence with HIV infection in poorer communities resulting in neuropathies [30].

### Cognitive function in HIV positive Bell’s palsy patients

There were no cognitive deficits detected in our patients with Bell’s palsy, including the HIV positive group. A literature search on HIV positive Bell’s palsy and cognitive function did not yield results. Future studies with robust assessments for even mild cognitive changes in HIV positive Bell’s palsy individuals could be of value. The high number of individuals in this study not on antiretroviral therapy is concerning. This however, would have improved post September 2016 with the introduction of the “test and treat” policy in South Africa [31]. The effect of antiretroviral therapy in HIV positive individuals has been shown not to grant absolutely protection of cognitive function. The reason for the latter finding is still a matter for research [32].

#### Clinical implications and perspectives for public

This study availed an opportunity for an improved understanding of incident Bell’s palsy and its risk factors in the black African patient. Prevention strategies could possibly emanate from improved knowledge of Bell’s palsy, a condition which continues to be regarded as idiopathic. Furthermore, efficacious and cost-effective treatment programmes will emerge from an in-depth understanding of Bell’s palsy. Bell’s palsy is also a possible outcome of psychological stressors linked with a poor socio-economic status prevalent amongst the studied population of black South Africans. Further studies on the latter are needed. Government legislation has since changed in South Africa, with much improvement in availability of ARV treatment.

## Conclusion

Environmental factors by seasonality, younger age and HIV positivity are important and independent risk factors of Bell’s palsy. Education and awareness programs on the possible effects of HIV and seasons on the development of Bell’s palsy are necessary. A better understanding of this condition and its predictors could lead to well designed preventative measures amongst young black South Africans.

## Data Availability

Data will be available on request to corresponding author.

## Abbreviations

HIV: Human immunodeficiency virus
HSV: Herpes simplex virus
DNA: Deoxyribonucleic acid
HR: Hazard ratio
RR: Relative risk
CI: Confidence interval
SPSS: Statistics package of social sciences
IBM: International business machines
US: United States
